# Angioimmunoblastic T‐cell lymphoma after acute myeloid leukemia: Alleged common pathogenesis. A case report and literature review

**DOI:** 10.1002/ccr3.3430

**Published:** 2020-11-03

**Authors:** Isabel Iturrate, Javier Loscertales, Elena Fernández‐Ruiz, Patricia Muñoz, Consuelo López, Luciana del Campo, Cecilia Muñoz, Adrián Alegre

**Affiliations:** ^1^ Department of Hematology Universitary Hospital de La Princesa Madrid Spain; ^2^ Department of Molecular Biology Universitary Hospital de La Princesa Madrid Spain; ^3^ Department of Pathological Anatomy Universitary Hospital de La Princesa Madrid Spain; ^4^ Department of Immunology Universitary Hospital de La Princesa Madrid Spain

**Keywords:** angioimmunoblastic T‐cell lymphoma, DNMT3A, epigenetic modifiers, myeloid malignancies, TET2

## Abstract

The genomic landscape of AITL is characterized by mutation of epigenetic modifiers. This gene expression pattern resembles myeloid diseases and shows a potential role for hypomethylating agents as possible therapy for AITL.

## Graphical abstract

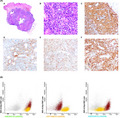

## 1 INTRODUCTION

Angioimmunoblastic T‐cell lymphoma (AITL) is one of the most frequent peripheral T lymphoma. This disease is associated with poor prognosis with a median survival of <3 years, without improvement with conventional therapies.^1^ Using next‐generation sequencing (NGS), the gene expression pattern of AITL was recently defined and is different from other T‐cell lymphomas. The genomic landscape of AITL is characterized by frequent mutation of epigenetic modifiers, including *TET2* (Ten‐Eleven Translocation 2), *DNMT3A* (DNA methyltransferase 3A), and *IDH2* (Isocitrate Dehydrogenase 2).^2^ These mutations resemble myeloid diseases more than other lymphomas, and they could, therefore, be considered as new therapeutic targets.

## 2 CASE PRESENTATION

Here, we describe the presence of angioimmunoblastic T‐cell lymphoma (AITL) in a 75‐year‐old woman who 10 years ago had acute myeloid leukemia (AML) with mutation in the gene encoding nucleophosmin 1 (*NPM1*).

In 2008, she was diagnosed with AML (FAB M5), *MLL*‐ (mixed‐lineage leukemia) negative, *NPM1*‐positive, normal karyotype, with infiltration of central nervous system. The patient achieved a complete remission (CR) after 2 cycles of cytarabine and anthracycline given in the standard 3 + 7 regimen, and posterior intermediate‐dose cytarabine. She also received six doses of triple intrathecal chemotherapy: cytarabine, methotrexate, and hydrocortisone. Subsequently, the patient underwent autologous stem cell transplantation with busulfan and cyclophosphamide (BuCy) as conditioning regimen. The patient remained in remission since then, with a very good quality of life and without any other therapy.

In 2016, the patient started with low platelet count at about 80 000 to 100 000/mm^3^, so a bone marrow aspiration was done. The patient was diagnosed with myelodysplastic syndrome with multilineage dysplasia secondary to previous chemotherapy (therapy‐related myeloid neoplasm by WHO), with del 5q associated. From 2016 to 2019, her disease remained stable. She was asymptomatic, did not receive any treatment, and no transfusion support was needed.

**Figure 1:**
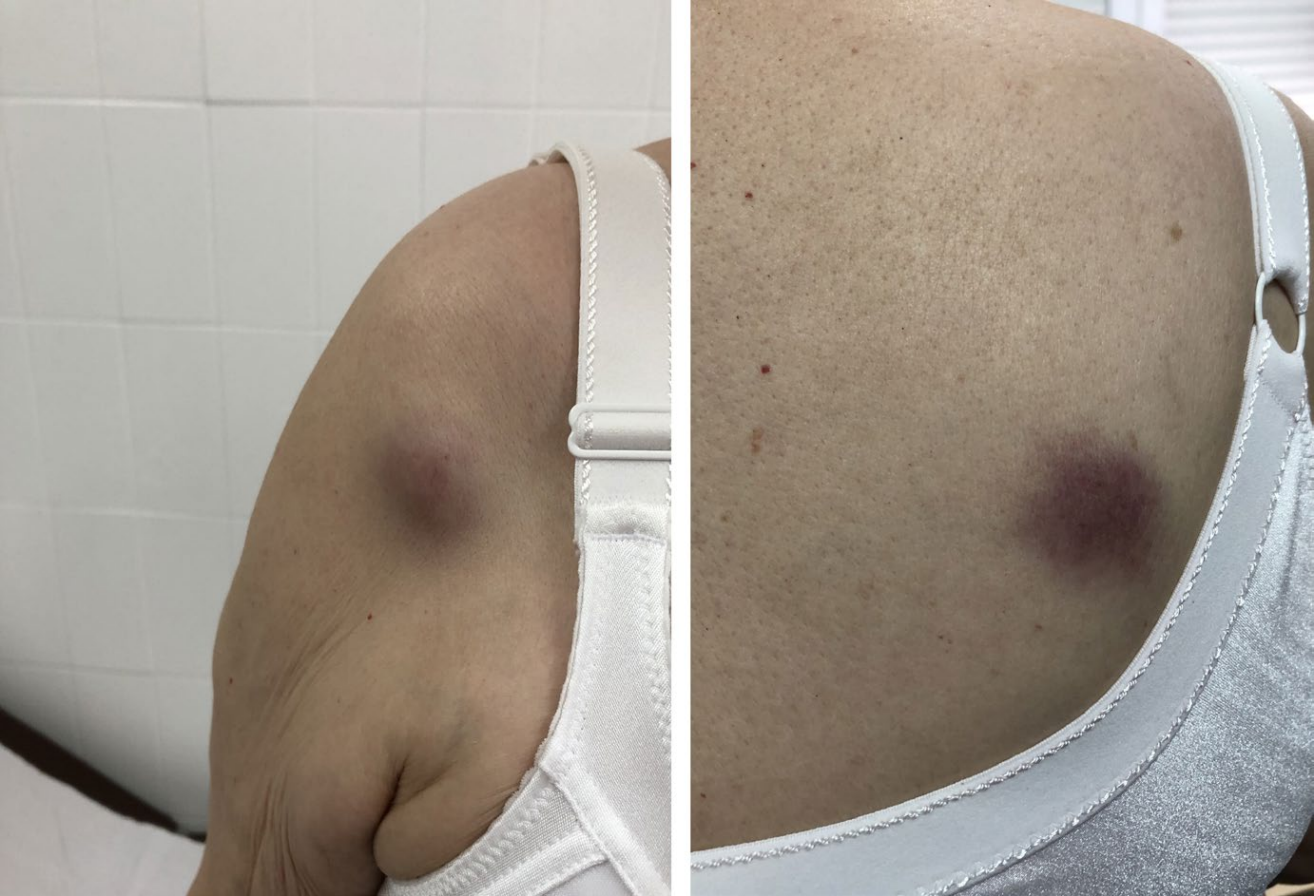
Skin lesions

In 2019, she presented with a 2‐month history of progressive lymphadenopathy in right axilla associated with skin lesions of the type purple infiltrative nodules of 2‐3 cm large in thoracic region (Figure 1). She did not have hepatoesplenomegaly nor B symptoms. She had mild anemia and thrombopenia, with normal LDH and no hypergammaglobulinemia. PET‐CT scan showed abnormal activity in the skin extending from the right arm through both supraclavicular regions, the back, and the lumbar region. It also showed intense and homogeneous hypermetabolic activity (SUV 14) at both cervical ganglionar regions, right axilla and right paratracheal region. There was hypermetabolic lymphadenopathy in right groin, with several lymph nodes, 40 mm in longest diameter (SUV 21). There was no hepatic or splenic involvement.

**Figure 2:**
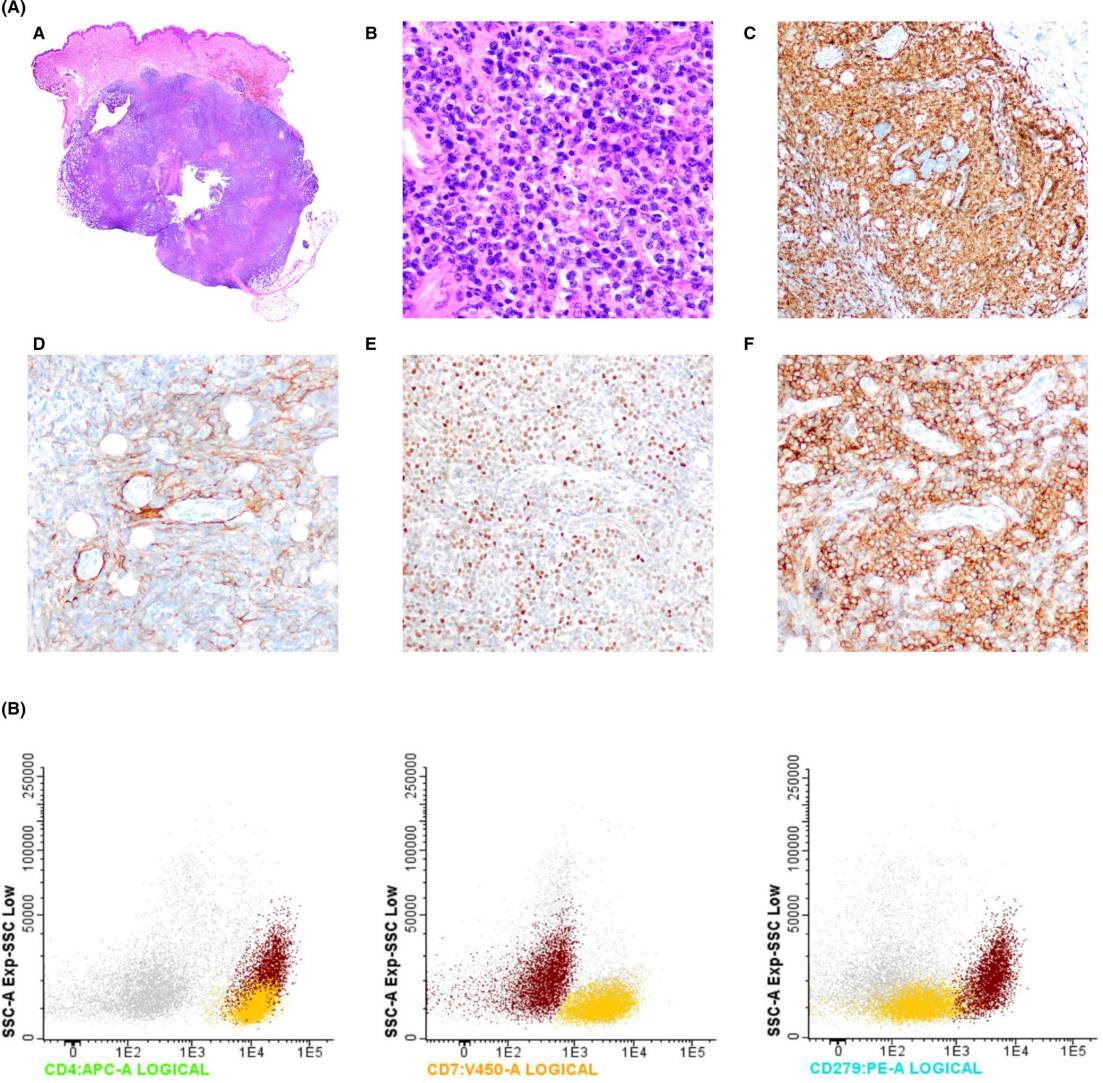
A, Histopathologic and immunophenotypic features of cutaneous involvement by angioimmunoblastic T‐cell lymphoma. (a) Panoramic view of a dense lymphoid infiltrate seen in dermis and subcutaneous tissue. H&E. (b) Small to medium neoplastic cells intermixed with vascular hyperplasia in a polymorphous inflammatory background. ×40, H&E. (c) Neoplastic lymphocytes are diffusely positive for CD3. ×10. (d‐f) Neoplastic T cells are variably positive for CD10 (d), BCL6 (e), and PD1 (f), TFH markers. ×20. B, The immunophenotypic analysis. Aberrant T‐cell population CD4+, CD7−, and CD279+ shown in red and normal T helper cells CD4+, CD7+, and CD279− in yellow

The skin biopsy showed a diffused dense infiltrate of small to medium‐sized neoplastic lymphocytes with irregular nuclei, evident nucleoli, and occasional clear cytoplasm located in the dermis and subcutaneous tissue. There was associated vascular hyperplasia with prominence of endothelial cells. The neoplastic infiltrate was accompanied by a polymorphous inflammatory background composed of reactive lymphocytes, plasma cells, and histiocytes. Tumor cells expressed pan‐T‐cell antigens (CD3 and CD5) and showed the immunophenotype of normal T follicular helper (TFH) cells (CD10, BCL6, and PD1). There were focal expanded follicular dendritic cell networks highlighted by CD21 and also Epstein‐Barr virus (EBV)‐positive cells. These EBV‐positive cells are typically B cells, often with CD30 expression, and are seen in nearly 95% of nodal AITL cases.^3^ The histologic diagnosis was cutaneous involvement of T‐cell lymphoma with TFH phenotype suggesting angioimmunoblastic T‐cell lymphoma (AITL). Figure 2A.

Eight‐color flow cytometry on skin biopsy was positive for an aberrant T‐cell population (29% of total white blood cells) of higher side scatter (SSC) than regular T cells and with the following markers: CD45+, CD3+, CD4+, CD8‐CD5+, CD279+, CD10dim+, and CD7−. Figure 2B.

In the bone marrow biopsy, there was no evidence of infiltration by lymphoma. Molecular studies of the bone marrow identified a clonal *TET2* somatic mutation. The *TET2* mutation was present in virtually almost all the bone marrow cells (variant allele frequency [VAF], 43.13%). In addition to this epigenetic modifier, *MLL* and *NPM1* were also mutated.

The patient is currently in complete remission after six cycles of chemotherapy with cyclophosphamide, doxorubicin, prednisone, and brentuximab vedotin.^4^ The follow‐up after completion of therapy is only of 2 months.

## 3 LITERATURE REVIEW AND DISCUSSION

AITL is an aggressive lymphoma derived from T‐cell follicular helper cells. Recent studies have shown that pathophysiology and genetics of AITL are different from other T‐cell lymphomas. This demonstrates an association of AITL with mutations in epigenetic regulators (*TET2*, *DNMT3A*, and *IDH2*) and with the expression of *RHOA* gene (Ras homolog family member A).^5^ Follicular T helper cells initially acquire premalignant mutations in *TET2* and *DNMT3A*, followed by mutations in *RHOA G17V* or *IDH2*.

The study by Odejide et al^2^ defined the first genetic landscape of AITL, describing *TET2* as the most frequent mutation, presenting in 76% of AITL. *DNMT3A* was the second most frequently mutated gene (33%) followed by *IDH2* (20%).^2^ *TET2* and *DNMT3A* have been described in myeloid neoplasms and in clonal haematopoiesis. In AITL, *TET2* mutations were found not only in the tumor cells but also in hematopoietic stem cells, suggesting that early *TET2* during hematopoiesis could lead to the development of AITL.^6^ In the present case study, *TET2* is mutated with a high VAF, which suggests an early event in pathogenesis.

It is also important to consider the use of busulfan in this patient, as it can cause genetic mutations and the development of second malignancies. Busulfan is commonly used in hematopoietic stem cell transplant setting as it has both myeloablative and antitumor properties. This alkylating drug is cytotoxic to proliferating tissues, and therefore, it can also cause several adverse events. The most common significant toxicity of busulfan is sinusoidal obstruction syndrome, neurotoxicity, and interstitial pneumopathies.^7^ Alkylating agents such as busulfan can cause second malignancies in which deletion of chromosome 5 and 7 is very frequent, and they usually appear 5‐10 years after primary cancer.

The development in the same patient of acute myeloid leukemia with mutation in *NPM1* and angioimmunoblastic T‐cell lymphoma with mutation in the *RHOA* gene was previously described in the literature.^8^ The common origin of these two hematologic neoplasms can be explained by a high‐risk CHIP (Clonal hematopoiesis of indeterminate potential) characterized by overlapping mutations in epigenetic modifiers such as *TET2*.^8^

## 4 CONCLUSION

The development of acute myeloid leukemia with mutation in *NPM1* and angioimmunoblastic T‐cell lymphoma, in the same patient, could be linked by a common pathogenesis.

Further studies are needed to elucidate the role of these genetic alterations in AITL pathogenesis and eventually develop molecularly targeted therapies to treat this aggressive disease.

## CONFLICT OF INTEREST

The authors have no conflicts of interest to declare.

## AUTHOR CONTRIBUTIONS

II, JL, and AA: analyzed and interpreted the patient data regarding the hematologic disease. EF: studied the mutations in epigenetic modifiers in the patient. PM and CL: performed the histologic examination of the skin lesions. LC and CM: performed flow cytometry on skin biopsy. All authors read and approved the final manuscript.

## ETHICAL APPROVAL

This research was conducted ethically in accordance with the World Medical Association Declaration of Helsinki. The patient has given the consent to publish her case.
